# Calreticulin: Challenges Posed by the Intrinsically Disordered Nature of Calreticulin to the Study of Its Function

**DOI:** 10.3389/fcell.2017.00096

**Published:** 2017-11-23

**Authors:** Lilian Varricchio, Mario Falchi, Massimiliano Dall'Ora, Caterina De Benedittis, Alessandra Ruggeri, Vladimir N. Uversky, Anna Rita Migliaccio

**Affiliations:** ^1^Tisch Cancer Institute, Icahn School of Medicine at Mount Sinai, New York, NY, United States; ^2^National HIV/AIDS Center, Istituto Superiore Sanità, Rome, Italy; ^3^Department of Biomedical and Neuromotorial Sciences, Alma Mater University, Bologna, Italy; ^4^Department of Molecular Medicine and USF Health Byrd Alzheimer's Research Institute, Morsani College of Medicine, University of South Florida, Tampa, FL, United States; ^5^Laboratory of New Methods in Biology, Institute for Biological Instrumentation, Russian Academy of Sciences, Pushchino, Russia

**Keywords:** calreticulin, glucocorticoid receptor, erythropoietin receptor, stress erythropoiesis, myeloproliferative disorders, intrinsically disordered proteins

## Abstract

Calreticulin is a Ca^2+^-binding chaperone protein, which resides mainly in the endoplasmic reticulum but also found in other cellular compartments including the plasma membrane. In addition to Ca^2+^, calreticulin binds and regulates almost all proteins and most of the mRNAs deciding their intracellular fate. The potential functions of calreticulin are so numerous that identification of all of them is becoming a nightmare. Still the recent discovery that patients affected by the Philadelphia-negative myeloproliferative disorders essential thrombocytemia or primary myelofibrosis not harboring JAK2 mutations carry instead calreticulin mutations disrupting its C-terminal domain has highlighted the clinical need to gain a deeper understanding of the biological activity of this protein. However, by contrast with other proteins, such as enzymes or transcription factors, the biological functions of which are strictly defined by a stable spatial structure imprinted by their amino acid sequence, calreticulin contains intrinsically disordered regions, the structure of which represents a highly dynamic conformational ensemble characterized by constant changes between several metastable conformations in response to a variety of environmental cues. This article will illustrate the Theory of calreticulin as an intrinsically disordered protein and discuss the Hypothesis that the dynamic conformational changes to which calreticulin may be subjected by environmental cues, by promoting or restricting the exposure of its active sites, may affect its function under normal and pathological conditions.

## Introduction

Calreticulin (CALR) is a Ca^2+^-binding protein expressed by all the cells and present in all their compartments, which, as implied by its name, has the major function to bind, buffer, and control intracellular Ca^2+^ levels (Wang et al., 2012). For long time, the functions of CALR were studied by a restricted group of scientists interested in defining the role of Ca^2+^ signaling in the control of the cellular response to stress. These studies, however, quickly identified that in the endoplasmic reticulum (ER), CALR binds not only Ca^2+^, but also interacts with many ER proteins, transiently associates with numerous different newly synthesized glycoproteins as they enter the secretory pathway (Peterson et al., 1995; Helenius et al., 1997; High et al., 2000), and even interact with some mRNAs, thereby deciding their fate (Michalak et al., 2009). Among established binding partners of CALR are the E3 ubiquitin-protein ligase TRIM21 (tripartite motif-containing protein 21, also known as 52 kDa ribonucleoprotein autoantigen Ro/SS-A; Cheng et al., 1996), the glucocorticoid receptor (GR, also known as the nuclear receptor subfamily 3 group C member 1, NR3C1; Holaska et al., 2001), PPIB (peptidyl-prolyl cis-trans isomerase B, also known as cyclophilin B; Kozlov et al., 2010a), GABARAP (Gamma-aminobutyric acid receptor-associated protein; Thielmann et al., 2009), PDIA3 (protein disulfide-isomerase A3, also known as endoplasmic reticulum resident protein 57, ERp57; Panaretakis et al., 2008), PDIA5 (protein disulfide-isomerase A5; Vinaik et al., 2013), and SPACA9 (sperm acrosome-associated protein 9, also known as C9orf9, or Acrosome and sperm tail protein; Bhattacharya et al., 2013). Furthermore, CALR serves as a component of the EIF2 (eukaryotic initiation factor 2) complex which also contains the CUG triplet repeat RNA-binding protein 1 (CUGBP1, also known as CUGBP Elav-like family member 1, CELF1), Calreticulin-3 (CALR3), eukaryotic translation initiation factor 2 subunits 1 and 2 (EIF2S1 and EIF2S2), heat shock protein 90 kDa beta member 1 (HSP90B1, also known as endoplasmin), and heat shock 70 kDa protein 5 (HSPA5, also known as 78 kDa glucose-regulated protein, GRP-78; Timchenko et al., 2006). The emerged functions of CALR are so numerous that it is impossible to describe all of them.

Great general interest for studying the full array of CALR functions has been recently raised by two clinical observations: namely, CALR was shown to be expressed on the cell membrane at levels which are altered in a variety of cancers (Chao et al., 2010; Wemeau et al., 2010), and indel or deletion mutations in exon 9 of *CALR* are associated with the majority of Philadelphia-negative myeloproliferative neoplasms which do not harbor mutations in *JAK2* (Klampfl et al., 2013; Nangalia et al., 2013; Nunes et al., 2015). These findings have inspired numerous studies on the normal and pathological function of CALR that up to now have used the well-established array of experimental approaches which have been instrumental to characterize the biological functions of many other proteins. However, by contrast with many of the commonly studied proteins which are characterized by well-defined 3D-structures, CALR is an intrinsically disordered protein or, more correctly, a hybrid protein, containing ordered domains and disordered regions, and does not have a unique 3D structure for significant part of its sequence (Shivarov et al., 2014; Migliaccio and Uversky, 2017). This fact poses novel challenges to biochemical and molecular biology investigators. This Hypothesis and Theory article will summarize CALR features that are “unique” due to its intrinsically disordered nature and hypothesize how these features affect our understanding of the biological and clinical implications of the protein.

## Structural features of normal human CALR

### Primary and secondary structure

**Figure 1:**
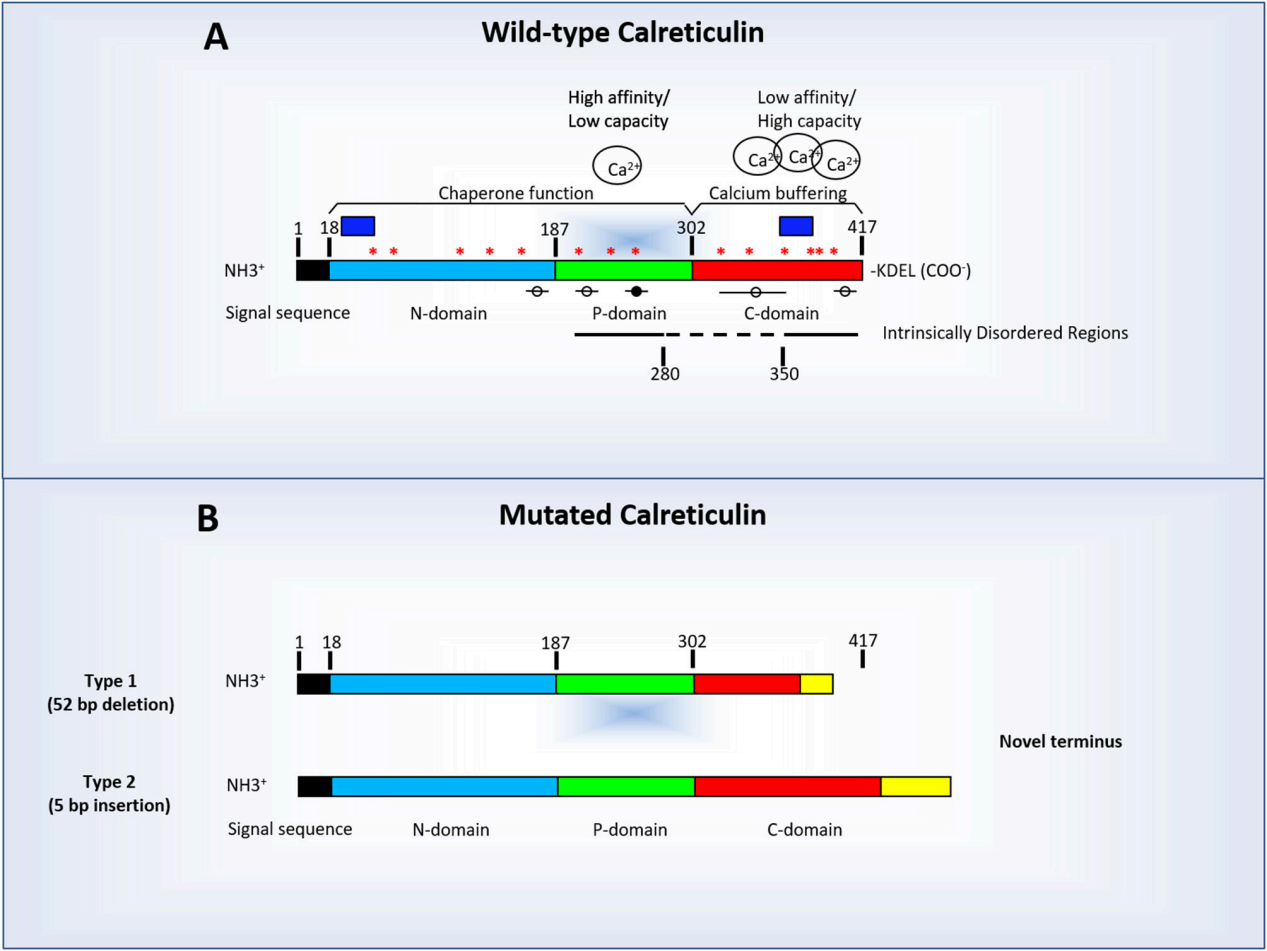
Linear diagram of human calreticulin indicating its amino-terminal (N-CALR), globular (P-CALR), and carboxy-terminal (C-CALR) domains and the location of the sequences predicted by computer modeling to determine an intrinsically disordered structure (indicated by the straight lines). **(A)** Normal human CALR and factors which may potentially affect its tertiary structure. In addition to the presence of sequences which determine the intrinsically disordered structure (straight lines), the tertiary structure of CALR is affected by the levels of Ca^2+^ bound to C-CALR and by binding of other proteins to putative MoRFs sequences (circles and lines). The black circle indicates MoRF3 which has a known putative binding protein. The dashed line indicate the region between AA 260–330 AA predicted by computer modeling to have a stable conformation (i.e., lacking intrinsically disordered regions, binding sites for Ca^2+^ or MoRFs). The blue boxes indicate the location of the sequences used to raise the commercially available antibodies against human N-CALR (#12238, Cell Signaling, Boston, MA) and C-CALR (sc-6467, Santa Cruz Biotechnology, Santa Cruz, CA). Asterisks indicate putative JAK2-dependent phosphorylation sites. **(B)** Diagram of the structure of representative Type 1 (deletions) and Type 2 (insertions) *CALR* mutations found in Philadelphia-negative myeloproliferative disorders. The mutations found in these maladies are all localized in exon 9 encoding the terminal C-region of the protein and encode a truncated (Type 1, top diagram) or elongated (Type 2, bottom diagram) form of C-CALR. In both cases, the mutated C-CALR loses the KDEL motive necessary for translocation in the ER. The mutations induce also loss of sites in the C domain responsible for Ca^2+^ binding, of three of the putative JAK2-dependent phosphorylation sites and two MoRFs sites (see Table 1 for further details). The mutant proteins may also lose the sequence used to generate the anti-C-CALR antibody commercially available. In this figure, as in rest of the manuscript CALR AA are numbered starting from the first AA of the signal sequence.

CALR is synthesized in the form of a precursor protein containing an N-terminally located signal peptide (residues 1–17). To avoid confusion, CALR residues will be numbered according to the amino acid (AA) sequence of the pre-protein (Figure 1). Mature CALR has a predicted molecular weight of 46 kDa and can be divided into three domains: the N-terminal (N-CALR, residues 18–197 in the UniProt ID: P27797), the proline-rich (P-domain, residues 198–308), and the C-terminal (C-CALR, residues 309–417) domains (Michalak et al., 1999; Figure 1).

N-CALR is encoded by a highly conserved AA sequence that is folded in a stable globular structure with eight antiparallel β-strands (Michalak et al., 1999, 2009). N-CALR is an important functional domain that includes polypeptide- and carbohydrate-binding sites (Leach et al., 2002; Kapoor et al., 2004), a Zn^2+^-binding site (Baksh et al., 1995), and a disulfide-linkage site (Andrin et al., 2000).

P-CALR consists of an extended region stabilized by three antiparallel β-strands (Ellgaard et al., 2001a), which includes three consecutive repeats of two AA sequences (PXXIXDPDAXKPEDWDE and GXWXPPXIXNPXYX, respectively) that have been associated with the lectin-like chaperone function of the protein (Michalak et al., 2009). P-CALR binds Ca^2+^ with high affinity (K_d_ = 1 μM), but low capacity (1 Ca^2+^ per protein; Michalak et al., 1999, 2009).

C-CALR has a highly acidic AA composition and is responsible for binding over 50% of the Ca^2+^ present in the ER lumen (Nakamura et al., 2001) with high capacity and low affinity (up to ~20 ions of Ca^2+^ per 1 molecule of CALR, K_d_ ~2 mM; Michalak et al., 2009). C-CALR exerts the Ca^2+^-buffering functions of the protein (Baksh and Michalak, 1991) and contains the KDEL (Lys-Asp-Glu-Leu) signal required for protein translocation across the ER membranes (Michalak et al., 2009).

CALR does not contain neither a transmembrane domain nor a GPI-anchor attachment site and instead is anchored to the membranes via specific adaptors, such as CD59 in neutrophils (Ghiran et al., 2003), or the receptors for thrombopoietin, erythropoietin or granulocyte colony stimulating factor (G-CSF) in other hematopoietic cells (Araki et al., 2016; Chachoua et al., 2016).

### Tertiary structure and intrinsic disorder

The beginning of the twenty-first century saw a paradigm shift in our understanding of the 3D-structure of a protein (Dunker et al., 2001; Uversky and Dunker, 2012). It became clear that under physiological conditions the primary sequence of many proteins is not sufficient to determine a unique 3D-structure. As a result, such proteins often contain regions of structural instability (defined as intrinsically disordered regions) or do not have ordered structure as a whole (are intrinsically disordered). Such intrinsically disordered nature defines the highly flexible character of such proteins that exist as dynamic conformational ensembles, which allows them to continuously shift among several possible conformations in response to environmental cues. In other words, the structure of the protein is in a dynamic equilibrium among multiple discrete conformational states. Several computer programs exist which are dedicated to the discovery of the intrinsically disordered regions of a protein and to the functional characterization of such regions (e.g., finding of the disorder-based binding sites).

**Figure 2:**
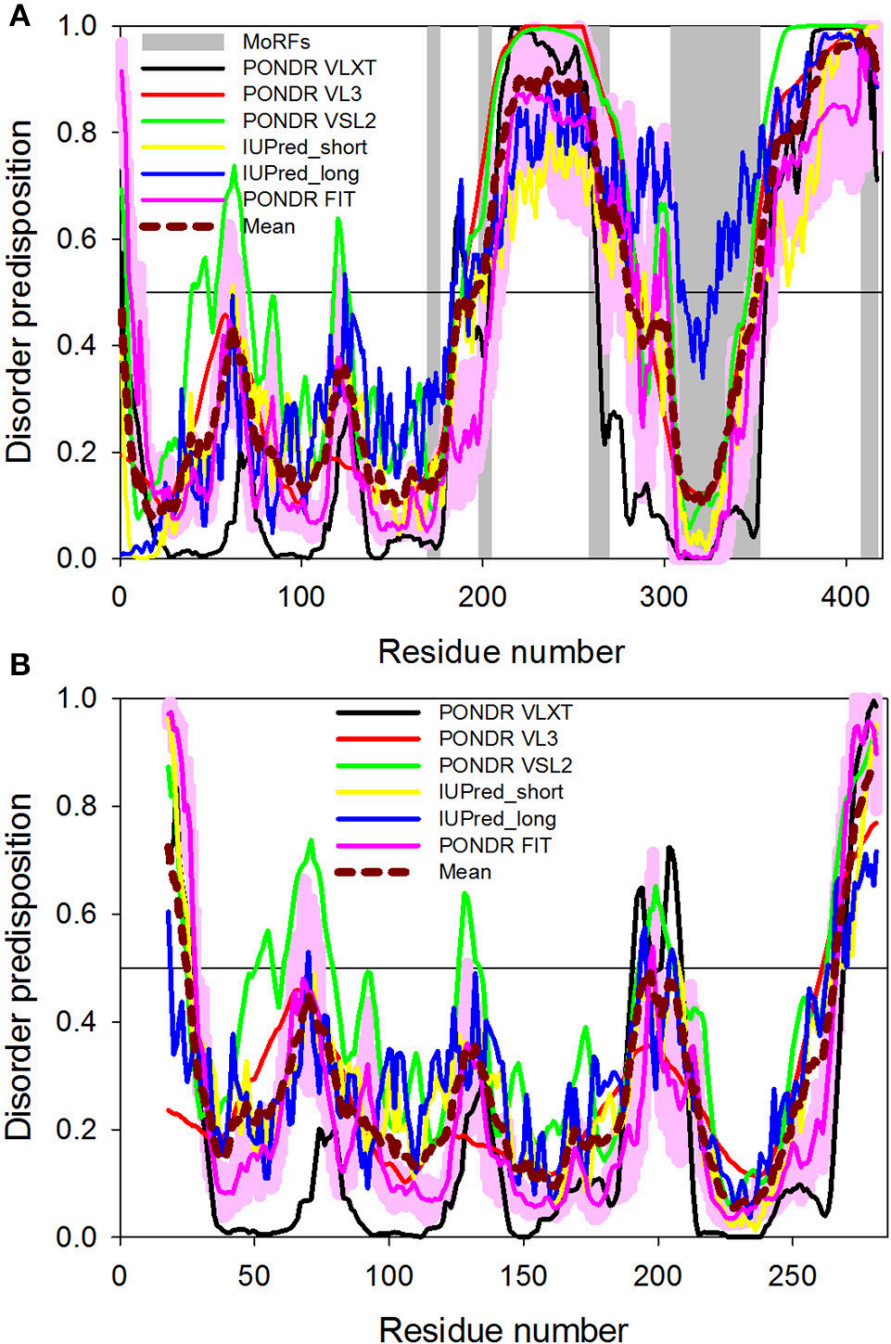
Intrinsic disorder propensity of wild-type human CALR **(A)** and of the construct of human CALR used in the crystallization study. **(A)** Evaluating intrinsic disorder propensity of human CALR (UniProt ID: P27797) by series of per-residue disorder predictors. Disorder profiles generated by PONDR® VLXT, PONDR FIT, PONDR® VL3, PONDR® VSL2, IUPred_short, and IUPred_long are shown by black, red, green, yellow, blue, and pink lines, respectively. Dashed dark red shows the mean disorder propensity calculated by averaging disorder profiles of individual predictors. Light pink shadow around the PONDR® FIT shows error distribution. In these analyses, the predicted intrinsic disorder scores above 0.5 are considered to correspond to the disordered residues/regions. Positions of the MoRF regions are shown as gray shaded areas. Modified from Migliaccio and Uversky (2017). **(B)** Intrinsic disorder propensity of the crystallized construct of human CALR described in Figure 3A by series of per-residue disorder predictors. The amino acid sequence of this construct includes the N-domain (residues 18–204) and the N-terminal half of the C-domain (residues 303–368) connected by a GSG tripeptide. Disorder profiles generated by the same program described in **(A)** [see legend of **(A)** for further details]. See Migliaccio and Uversky (2017).

The intrinsic disorder predisposition of CALR was identified by two independent multiparametric computational analyses (Shivarov et al., 2014; Migliaccio and Uversky, 2017), which utilized, respectively, either PONDR-FIT (Xue et al., 2010) and RONN (Yang et al., 2005) disorder predictors (Shivarov et al., 2014) or four different algorithms from the PONDR family (Li et al., 1999; Romero et al., 2001; Obradovic et al., 2005; Peng et al., 2005, 2006; Xue et al., 2010) and the IUPred web server (Dosztányi et al., 2005; Migliaccio and Uversky, 2017). These analyses revealed that the N-domain was expected to be mostly ordered, whereas the P-domain and the C-terminal tail of C-domain were highly disordered. Figure 2A represents results of the analogous computational analysis, where the per-residue intrinsic disorder propensity of human CALR is evaluated by PONDR-FIT (Xue et al., 2010), PONDR® VLXT (Dunker et al., 2001), PONDR® VSL2 (Obradovic et al., 2005), and PONDR® VL3 (Obradovic et al., 2003), as well as by the IUPred web server with its two versions for predicting long and short disordered regions (Dosztányi et al., 2005). These computational tools were selected because of their specific features. PONDR® VSL2 is one of the most accurate stand-alone disorder predictors (Obradovic et al., 2005), PONDR® VL3 possesses a high accuracy for finding long disordered regions (Obradovic et al., 2003), PONDR® VLXT is not the most accurate predictor, but has a high sensitivity to local sequence peculiarities which are often associated with disorder-based interaction sites (Dunker et al., 2001), PONDR-FIT is a metapredictor that is moderately more accurate than each of the component predictors and that is one of the most accurate disorder predictors (Xue et al., 2010), whereas IUPred utilizes the pair-wise energy estimation approach for finding intrinsically disordered residues/regions and can be used for finding both short and long disordered regions (Dosztányi et al., 2005). We also evaluated the consensus disorder propensity of human CALR by averaging disorder profiles of individual predictors. The use of consensus for evaluation of intrinsic disorder was motivated by empirical observations that the predictive performance can be increased by this approach compared to the use of a single predictor (Peng and Kurgan, 2012; Fan and Kurgan, 2014; Walsh et al., 2015). Figure 2A clearly shows that human CALR is expected to have two long disordered regions, residues 196–280 and 350–417 corresponding to the large portion of P-CALR and the acidic C-tail, respectively.

**Figure 3:**
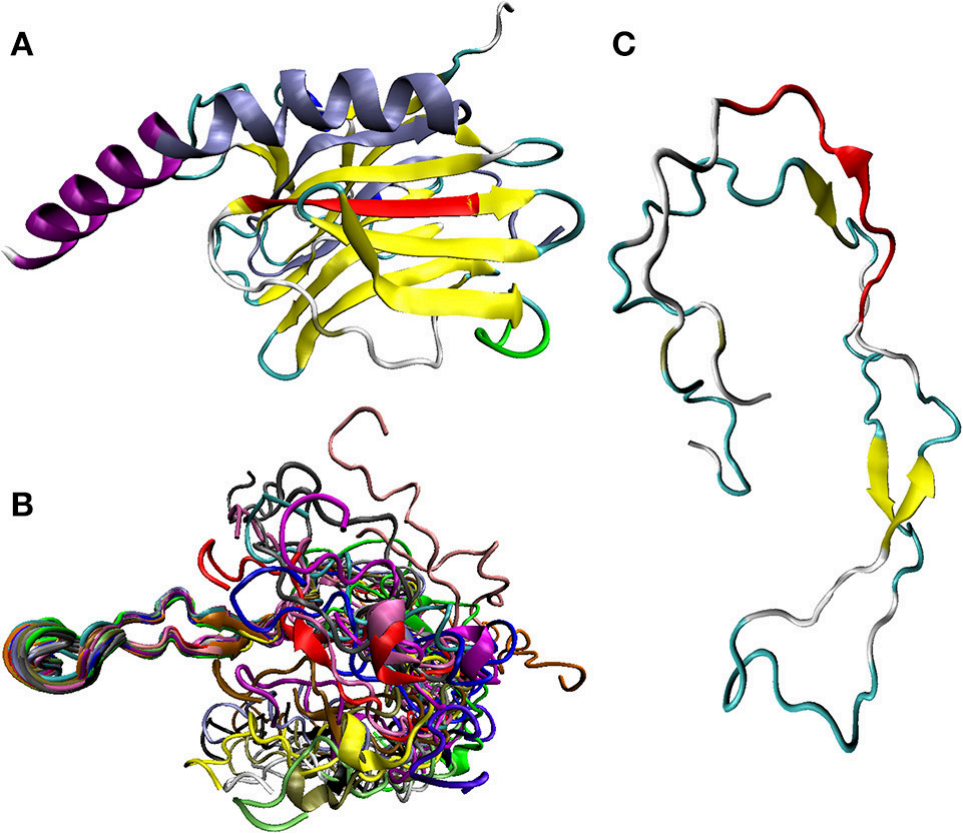
Structural characterization of human and rat CALR. **(A)** X-ray crystal structure of the N-domain human of CALR (residues 18–204) and the N-terminal half of the C-domain (residues 303–368) connected by a GSG tripeptide (PDB ID: 3POW; Chouquet et al., 2011). The expected localization of MoRF1 (residues 170–176, in red), MoRF2 (residues 198–204, green), and MoRF4 (residues 304–352, ice-blue) in the crystal structure of the human protein is indicated. Note that in this construct, the P-domain is removed and replaced by the GSG tripeptide. With the exception to MoRFs that have their own color coding (see above), CALR structure is colored according to the secondary structure content, with β-strands, α-helices, 3_10_ helices, β-turns, and irregular structure shown by yellow, purple, blue, cyan, and white colors, respectively. **(B)** Solution NMR structure of the P-domain of the rat CALR (residues 206–305 in UniProt ID: P18418) (PDB ID: 1HHN; Ellgaard et al., 2001b). NMR analysis generates an ensemble of structures, all of which are consistent with the observed experimental restraints. Shown here is a conformational ensemble representing structure of the P-domain, which is one of the regions removed from the construct used in the crystallization studies of the human protein (see Figure 3A). This conformational ensemble contains an overlay of 20 molecular models, each shown by its own color. A highly dynamic nature of the conformational ensemble describing structure of P-CALR is evidenced by the “fuzzy” appearance of the conformational ensemble describing its solution structure. **(C)** Solution NMR structure of one of the members of the P-domain dynamic conformational ensemble (model 1 in PDB ID: 1HHN; Ellgaard et al., 2001b). Position of MoRF3 (residues 259–269) is indicated in red. With the exption to this MoRF, structure of P-CALR is colored according to the secondary structure content, with β-strands, β-turns, and irregular structure shown by yellow, cyan, and white colors, respectively.

The presence of potential disorder-based protein binding sites in human CALR was evaluated by the ANCHOR algorithm (Dosztányi et al., 2009). This algorithm utilizes the pair-wise energy estimation approach originally used by IUPred (Dosztányi et al., 2005). This approach is based on the hypothesis that long disordered regions might include localized potential binding sites, which are not capable of folding on their own due to not being able to form enough favorable intrachain interactions, but can obtain the energy to stabilize via interaction with a globular protein partner (Dosztányi et al., 2009). This ANCHOR analysis revealed that the intrinsically disordered regions of CALR contain five putative disorder-based protein binding sites (molecular recognition features, MoRFs) that can fold as a result of the interaction with specific partners (residues 170–176, 198–204, 259–269, 304–352, and 409–417; Figure 2A and Table 1), suggesting that these regions can be used for promiscuous protein-protein interactions. The five MoRFs are:

- MoRF1: a β-strand in the core structure of CRT, on the outer side interacting with the C-terminal α-helix (shown in red in Figure 3A).
- MoRF2: The initial part of the P-arm that functions as a switch between the closed and open conformation in parisite CALRs (Moreau et al., 2016; shown in green in Figure 3A).
- MoRF3: part of the down-stream of the P-arm (shown in red in Figure 3B).
- MoRF4: The inserted β-strands of the C-terminal region and the first part of the α-helix (shown in ice-blue in Figure 3A).
- MoRF5: The tip of the C-terminal region (containing the KDEL sequence; not present in the crystallized construct presented in Figure 3).

**Table 1:** Effects of the CALR mutations identified so far in patients with myeloproliferative neoplasm on the intrinsic disorder-based interactivity of the protein.

**Mutation**	**MoRF1**	**MoRF2**	**MoRF3**	**MoRF4**	**MoRF4′**	**MoRF5**	**MoRF5′**
**Wild type**	**170–176**	**198–204**	**259–269**	**304–352**		**409–417**	
**TYPE 1**							
p.E378Cfs^*^36	170–176	198–204	259–269	304–334		405–415	
p.K374Nfs^*^47	170–176	198–204	259–269	304–335		410–422	
p.D373Rfs^*^48	170–176	198–204	259–269	304–335		409–422	
p.D373Gfs^*^53	170–176	198–204	259–269	304–335	384–392		413–427
p.D373Vfs^*^57	170–176	198–204	259–269	304–334			416–428
pD373Afs^*^51	170–176	198–204	259–269	304–334	382–388		411–425
p.L367Tfs^*^46	170–176	198–204	259–269	304–334		401–414	
p.Q368Rfs^*^47	170–176	198–204	259–269	305–333		401–414	
p.K360Wfs^*^49	170–176	198–204	259–269	305–333		398–410	
p.E370Vfs^*^43	170–176	198–204	259–269	304–334		399–411	
p.E370Qfs^*^48	170–176	198–204	259–269	304–334		406–419	
p.L367Qfs^*^48	170–176	198–204	259–269	304–334		403–416	
p.L367Rfs^*^44	170–176	198–204	259–269	305–334		398–411	
p.K368Rfs^*^51	170–176	198–204	259–269	304–334		406–420	
p.L367Rfs^*^52	170–176	198–204	259–269	304–334	378–384	406–420	
p.R366Kfs^*^53	170–176	198–204	259–269	304–334	378–385	406–420	
p.E371Rfs^*^49	170–176	198–204	259–269	304–334		407–421	
p.K368Mfs^*^43	170–176	198–204	259–269	305–333		400–412	
p.E370Rfs^*^37	170–176	198–204	259–269	304–334		394–405	
p.E373Rfs^*^47	170–176	198–204	259–269	304–334		408–421	
p.K374Rfs^*^53	170–176	198–204	259–269	304–335	385–393		414–428
p.E371Rfs^*^49	170–176	198–204	259–269	304–334		406–419	
**TYPE 2**							
p.K385Nfs^*^47	170–176	198–204	259–269	304–338	388–394		418–430
p.K385Lfs^*^47	170–176	198–204	259–269	304–337	385–393		418–430
**COMPLEX**							
p.K376Lfs^*^55	170–176	198–204	259–269	304–334	389–397		419–432
p.K385Sfs^*^47	170–176	198–204	259–269	304–338	388–396		420–433
p.E381Dfs^*^48	170–176	198–204	259–269	304–337	386–393		417–430

*Expected effects of indel mutations on the interactivity of CALR were evaluated by the ANCHOR algorithm (Dosztányi et al., 2009; Mészáros et al., 2009). To this end, the AA sequence of each mutant (column 1) was analyzed by ANCHOR and the corresponding outputs (positions of MoRFs) were tabulated. Some of the indel Type 1 mutations and all the deletion Type 2 mutations induce formations of novel MORFs indicated in the column MoRF4′ and MoRF5′*.

Based on the crystallografic analysis of a construct containing the N-domain (residues 18–204) and the N-terminal half of the C-domain (residues 299–368) of human CALR connected by a GSG tripeptide but lacking the entire P-domain and C-terminal tail of the C-domain (see below), MoRF1 and MoRF4 are integral parts of the core region of CALR and unlikely to change conformation unless the entire core region is rearranged. Although, the core retains its overall structure both in the presence and absence of Ca^2+^ (Boelt et al., 2016), this possibility is theoretically possible and deserves to be further investigated. Since MoRF2 is located in a region that serves as a switch between the closed and open conformation [at least in the CALR from two distinct parasites, *Trypanosoma cruzi* (TcCALR) and *Entamoeba histolytica* (EhCALR; Moreau et al., 2016)], this molecular recognition feature clearly deserves further analysis. However, both MoRF3 and MoRF5 are likely to be disordered in their unbound forms, as great mobility of the regions they are located within has been generally agreed upon and shown in many cases. The fact that MoRF3 and MoRF5 are those mostly affected by SNPs associated with cancer (see below) strongly suggests that they play relevant biological functions.

It is possible that the intrinsically disordered nature of CALR may determine which of these sites are exposed at any given moment, and therefore which protein can be engaged in CALR binding. On the other hand, binding of the protein partners to these regions may affect the 3D-structure of CALR, thereby ensuring a two-way regulation between the two partners.

Despite the general knowledge of high binding promiscuity of CALR (as aforementioned, this protein was shown to interact with many proteins and numerous partially folded substrates present in ER; Michalak et al., 2009), the only CALR partner with a known binding site identified so far is peptidyl-prolyl cis-trans isomerase B, which binds residues 237–270 (Kozlov et al., 2010a), a region overlapping with MoRF3 (residues 259–269).

An intrinsically disordered structure appears to be a common feature of proteins exerting chaperone functions. In fact, in addition to CALR, several studies have indicated that numerous protein and RNA chaperones are either disordered or contain intrinsically disordered regions needed for their functionality (Tompa and Csermely, 2004; Tompa and Kovacs, 2010; Kovacs and Tompa, 2012). These proteins include late embryogenesis abundant (LEA) and other stress-related plant proteins (Kovacs et al., 2008a,b; Cuevas-Velazquez et al., 2017), human chaperones engaged in neurodegenerative diseases (Uversky, 2011), histone chaperones (Liu et al., 2017; Warren and Shechter, 2017), a redox-regulated chaperone Hsp33 (Rimon et al., 2017), small heat shock proteins (Sudnitsyna et al., 2012), and the heat shock transcription factors (Westerheide et al., 2012).

The intrinsically disordered nature of long regions in CALR has challenged crystallization of the full-length human protein (Chouquet et al., 2011). As a result, X-ray crystallographic structure is currently available only for a construct of human CALR containing the N-domain (residues 18–204) and the N-terminal half of the C-domain (residues 299–368) connected by a GSG tripeptide but lacking the entire P-domain and C-terminal tail of the C-domain (PDB ID: 3POW; Chouquet et al., 2011). Similarly engineered constructs were also used to obtain crystal structures of mouse CALR (PDB ID: 3O0W; Kozlov et al., 2010b), as well as CALR from the parasite *T. cruzi* (PDB ID: 5HCF; Moreau et al., 2016). In these studies, mouse construct contained the N-domain (residues 18–206) connected by a short linker to the N-terminal half of the C-domain (residues 301–368), whereas the parasite construct included the N-domain (residues 22–207) and a shorter fragment of the N-terminal half of the C-domain (residues 338–362) also connected by a short linker. Since the resulting structures of the recombinant human, mouse, and Trypanosoma proteins were rather similar, only the crystal structure of the human construct is discussed below as an example. Figure 3A shows that the structure of human construct is characterized by a jelly-roll fold formed by one convex and one concave anti-parallel β-sheets (Chouquet et al., 2011). The part of the C-domain, which is integrated into the globular domain, provides two central strands to the β-sandwich structure and adds a long kinked C-terminal α-helix (Chouquet et al., 2011). These observations suggest that the overall CALR structure can be viewed as a globular body comprised of the N-domain and the N-terminal half of the C-domain (structure of which can be resolved crystallographically) with two flexible and functionally different arms corresponding to the P-domain (residues 198–308) and C-tail (residues 370–417; Michalak et al., 2009; Wijeyesakere et al., 2011). In agreement with this model, crystal structure of the lumenal domain of dog calnexin (residues 45–468 of the UniProt ID: P24643) that includes an entire intact P-arm (residues 277–410) showed that this protein (which is a type-I integral membrane protein serving as another lectin-like chaperone in the endoplasmic reticulum) contains a compact globular domain (residues 61–262, 415–458) with a structure characteristic of legume lectins; i.e., a β-sandwich of two antiparallel β-sheets, and a 145-residue long arm (residues 270–414) protruding 140 Å away from the globular domain (PDB ID: 1JHN; Schrag et al., 2001).

Curiously, application of the ANCHOR algorithm to the sequence of a human CALR used in the crystallization study revealed that all MoRFs predicted in the original natural protein were eliminated due to the removal of P-domain and C-terminal tail of the C-domain. This is rather unexpected consequence, since the analysis of the full-length CALR predicts that only MoRF3 (residues 259–269) and MoRF5 (residues 409–417) are removed from the chimeric protein used for crystallization (the CALR regions containing these MoRFs are not included in the crystallizable construct) while MoRF1 (residues 170–176) and MoRF2 (residues 198–204), which are located in the N-terminal domain, (residues 18–204), and MoRF4 (residues 304–352), which is contained in the N-terminal half of the C-domain (residues 299–368) should be retained. These considerations are exemplified by a comparison of Figures 2A,B which indicates that not only the overall disorder predisposition of CALR, but also its predicted disorder-based functionality are dramatically affected by removal of the P-domain and of the C-terminal tail of the C-domain. Although, MoRFs were not predicted by computer program in the sequence of the crystallized CALR construct, we marked the expected positions of MoRF1 (residues 170–176, red), MoRF2 (residues 198–204, green), and MoRF4 (residues 304–352, ice-blue) in the crystal structure of human protein presented in Figure 3A. Of these three MoRFs, only MoRF2, which is located in a loop region, is accessible to solvents, whereas MoRF1 serves as a β-strand located on the outer side of the core structure of CALR and interacts with the C-terminal α-helix and MoRF4 exists in the form of the inserted β-strands of the C-terminal region and the first part of the α-helix (Figure 3A). It is the observation that MoRF1 and MoRF4 are integral parts of the core region of the crystallized human CALR that leads to the conclusion that for them to undergo changes in conformation requires rearrangement of the entire core region. However, it is important to remember that this crystal structure was obtained with a construct which lacked the most flexible regions of the protein and contained only 3 residues instead than the almost 100 residues of the highly dynamic region of the P-domain. Since these sequence alterations may dramatically affect the structure and dynamics of CALR, the resolved X-crystallographic structure of this construct may not predict that of the functional protein.

Although, no detailed structural information is currently available for the P-domain of human CALR, the NMR solution structure was solved for the analogous region of the rat protein (residues residues 206–305 in UniProt ID: P18418) (PDB ID: 1HHN; Ellgaard et al., 2001b). Overall human and rat proteins have high level of sequence identity (97.5%), and also rat CALR is predicted to have five MoRFs located at positions almost identical to that of the corresponding MoRFs in the human protein (residues 171–176, 198–204, 259–269, 303–350, and 408–416 of the mature form of rat CALR). Since the AA sequences of the P-domains of human and rat CALR are almost identical, the structure determined for the rat P-domain may elucidate structural peculiarities of the human P-domain. Figure 3B shows that in rat CALR this region is characterized by an extremely high structural flexibility, which is evidenced by the “fuzzy” appearance of it structural ensemble containing 20 individual chains. To present a clearer view, Figure 3C represents the structure of a representative members of this dynamic conformational to illustrate that the P-domain is characterized by an unusual extended hairpin fold with high conformational flexibility (Ellgaard et al., 2001b). It was indicated that two very short β-strands, residues 224–226 and 248–250, constitute an anti-parallel β-sheet that closes the central loop (residues 227–247) of this domain that also contains two additional two-stranded antiparallel β-sheets comprising pairs of short β-strands, residues 207–209 and 262–264, and 190–192 and 276–278 (Ellgaard et al., 2001b). Figure 3C shows that MoRF3 (residues 259–269) includes one of the aforementioned short β-strands (residues 262–264). Recently, small-angle X-ray scattering (SAXS) analysis revealed that the intact human CALR is characterized by a highly asymmetric structure containing extended globular structure formed by the N- and C-terminal domains and a highly flexible P-domain (P-loop) protruding from the globular part, and with the acidic C-terminal tail occupying a volume opposite the P-loop relative to the core region (Nørgaard Toft et al., 2008). These SAXS results provided strong support to the notion that CALR is characterized by a highly dynamic structure containing globular core decorated with long flexible regions.

Subsequent biophysical studies added a novel twist to the characterization of the intrinsically disordered nature of CALR by indicating that the overall tertiary structure of this protein can be described in terms of metastable equilibrium between two conformational ensembles, which is sensitive to the Ca^2+^ concentration present in solution (Villamil Giraldo et al., 2010; Wijeyesakere et al., 2011; Migliaccio and Uversky, 2017). Ca^2+^ binding was shown to reduce the overall solvent accessibility of the hydrophobic core of the full-length CALR (measured as changes in the fluorescence parameters of the hydrophobic fluorescent probe ANS) and to increase its compaction. It also affected urea-induced unfolding of the protein, providing further support to the idea that Ca^2+^ shifts its conformational ensemble toward more folded conformations. The same studies, however, when conducted on isolated domains provided strikingly different results. In fact, Ca^2+^ concentrations did not affect neither the structure nor the conformation stability of the isolated P-domain while had some but minimal effects on that of the C-domain, indicating that the effects of Ca^2+^ on the intrinsically disordered nature of CALR are manifested only when the two domains are both present (Migliaccio and Uversky, 2017). Of note, all these biophysical studies were performed at physiologic Ca^2+^ concentrations (0.01–100 nM in the endoplasm, 50–500 μM in the ER) providing indication that the described structural changes may also occur *in vivo* (Villamil Giraldo et al., 2010; Wijeyesakere et al., 2011; Migliaccio and Uversky, 2017). The concept of physiological Ca^2+^ concentrations is rapidly evolving: intracellular Ca^2+^ concentrations in mammalian cells are in the 10–100 nM range at steady state and increase by 10- to100-fold (0.1–1 μM) upon stimulation. Since these concentrations are much lower than that present in human plasma [2 mM], it is possible that one of the functions of the structural changes of CALR is to prevent Ca^2+^ shocks when the cells are overstimulated. However, is spite of the important role exerted by CALR in controlling Ca^2+^ homeostasis *in vivo*, the levels of Ca^2+^ bound to this protein inside cells exposed to different conditions are still poorly characterized.

The high sensitivity of the CALR structure to Ca^2+^ concentration has potential functional importance, since several studies indicated that the chaperone activity of CALR is Ca^2+^-dependent (Wiuff and Houen, 1996; Rizvi et al., 2004; Conte et al., 2007). In agreement with these conclusions and observations, a careful analysis of the effect of Ca^2+^ binding on structural properties of the full-length mature CALR conducted by Boelt et al. using chemical cross-linking, mass spectrometry, bioinformatics analysis, and modeling in Rosetta revealed that the overall structure of this protein undergoes large Ca^2+^-induced changes (Boelt et al., 2016). In fact, the acidic C-terminal region of CALR was shown to move relative to the core in the Ca^2+^-dependent manner, and the highly flexible P-loop moved closer to the protein core and the acidic C-terminal tail following the Ca^2+^ binding (Boelt et al., 2016). These Ca^2+^-promoted structural changes causing relatively close interaction of the P-loop with the acidic C-terminal region were suggested to sterically occlude the lectin site/peptide binding site of CALR, thereby providing the first structural hint on the molecular mechanisms underlying the Ca^2+^-dependent regulation of CALR functionality (Boelt et al., 2016). The importance of the Ca^2+^-dependent interaction of P-CALR and acidic C-tail with the globular core for regulation of the chaperone function of this protein is further supported by the fact that the polypeptide binding conformation of CALR can be induced by heat shock, calcium depletion, or by deletion of the C-terminal acidic region (Rizvi et al., 2004).

Detailed structural analysis of CALR is complicated not only by the presence of long disordered regions, but also by the tendency of this protein to dimerize and self-oligomerize, a property that was shown to be important for the chaperone activity of CALR, since self-oligomerization noticeably increased CALR binding to peptides and denatured proteins (Jørgensen et al., 2003; Rizvi et al., 2004; Boelt et al., 2016) and also promoted its immunological activities (Huang et al., 2013; He et al., 2014). Obviously, any experimental technique that is used for the analysis of structural properties and conformational behavior of wholly disordered proteins can by applied for the investigation of isolated domains of CALR. Note that this “dissection” approach was successfully used in several previous studies dedicated to CALR that also discussed the intrinsically disordered nature of CALR and the intrinsic disorder status and high flexibility of the C- and P-domains of this protein (Bouvier and Stafford, 2000; Li et al., 2001; Nørgaard Toft et al., 2008; Yan et al., 2010; Wijeyesakere et al., 2011).

Although, the highly dynamic nature of the structure of intrinsically disordered proteins makes their structural analysis challenging, suitable techniques are being rapidly developed in numbers that are already approaching a few dozen (Uversky and Dunker, 2012; Uversky, 2015). These techniques are represented by a host of biophysical methods that provide information on the overall compactness, conformational stability, shape, residual secondary structure, transient long-range contacts, regions of restricted, or enhanced mobility, etc., of such proteins (Uversky and Dunker, 2012; Uversky, 2015). Because of their highly heterogeneous nature and conformational dynamics at multiple time-scales, the full spectrum of structural and dynamic characteristics of disordered proteins cannot be gained by a single tool and clearly requires multi-parametric approaches (Uversky and Dunker, 2012; Uversky, 2015). The structural characterization of the full-length CALR may also be tackled with experimental approaches such as a combination of chemical cross-linking with mass spectrometry (MS) and structure modeling (Back et al., 2003; Sinz, 2003, 2006; Jin Lee, 2008; Leitner et al., 2010; Peng et al., 2014; Sinz et al., 2015) which has already provided important information about CALR (Nielsen et al., 2007; Boelt et al., 2016). Other suitable techniques which may be used in the future are represented by heteronuclear multidimensional NMR, in-cell NMR, fluorescence resonance energy transfer (FRET), the H/D exchange analysis, limited proteolysis, a combination of SAXS with high-resolution NMR, atomic force microscopy-based single molecule force spectroscopy, high-speed atomic force microscopy, and single molecule FRET.

### Post-translational modification

CALR is synthesized as a precursor protein which undergoes proteolytic cleavage of its first 17 AA that are necessary to target the protein to the lumen of the ER. Once this N-terminal region is cleaved, CALR is accumulated in the ER thanks to the presence of the C-terminal KDEL retention motif. Computational analyses (http://ptmfunc.com/) predict that CALR may be subjected to several post-translational modifications. In fact, these programs have identified six putative ubiquitination sites (K48, K55, K143, K153, K159, and K206) and nine putative acetylation sites (K48, K62, K64, K153, K159, K206, K207, K209, and K238) in its N-CALR and P-CALR domains. Computer analyses (http://KinasePhos2.mbc.nctu.edu.tw/) also revealed the presence of 14 putative JAK2-dependent phosphorylation sites distributed along the entire length of the protein (Figure 1A). All these modifications have the potential to alter the 3D-structure and function of the protein. However, whether CALR is subjected at least to some level of post-translational modification has not been established as yet.

### Experimental challenges posed by the intrinsically disordered nature of CALR

Traditionally, protein biochemical studies involve size fractionation by electrophoresis in polyacrylamide gels containing urea and sodium dodecyl-sulfate (SDS-PAGE), which denature and linearize the proteins allowing their further identification with specific antibodies. SDS-PAGE has been optimized in order to efficiently disrupt all non-covalent interactions, to dissolve protein-based complexes, and to linearize the molecules, making all their part equally accessible to antibodies. However, intrinsically disordered proteins have been shown to be resistant to SDS-PAGE denaturation. Several of them may remain in a non-covalently bond state with other proteins upon SDS treatment and to run as complexes on SDS-PAGE (Tramentozzi et al., 2008; Shimada and Kitada, 2011; Das et al., 2012; Falson et al., 2015; Sadler et al., 2015). Furthermore, hydrophilic residues of disordered proteins and hybrid proteins containing ordered and disordered regions, such as the *Xenopus* XPA (Xeroderma pigmentosum group A) and the prostate-associated gene 4 protein, may bind SDS poorly, which allows them to migrate noticeably slower than the migration expected on the basis of their AA sequence (Iakoucheva et al., 2001; Tompa, 2002; Receveur-Bréchot et al., 2006; Zeng et al., 2011; Uversky and Dunker, 2012). As predicted by the highly disordered proline-rich domain and highly acidic C-tail of the protein and demonstrated more than 40 years ago, CALR binds SDS poorly and, therefore, is resistant to SDS-induced linearization. In addition, the aforementioned biophysical studies on the urea-induced unfolding of CALR and its isolated domains suggest that also the sensitivity of CALR to urea may be limited by the amount of Ca^2+^ bound to the protein, possibly as a reflection of the concentration of this ion in the cells being analyzed. The fact that CALR partially retain its 3D-structure in SDS-PAGE explains why CALR migrates in SDS-PAGE with an apparent molecular weight greater (55–60 KDa) than the theoretical 46 KDa expected on the basis of its AA sequence (Michalak et al., 1992; Klampfl et al., 2013; Nangalia et al., 2013; Falchi et al., 2017). Although, it is still unclear whether CALR undergoes post-translational modifications, all the aforementioned post-translational modifications have the potential to affect SDS binding at least to some of its domains allowing CALR to retain some globular structure.

As consequence of retention of tertiary structures, epitopes of the protein, which were poorly exposed *in vivo* may remain poorly exposed after SDS-PAGE separation, affecting their accessibility by antibodies in Western blot analyses. Of note, some of the currently available commercial antibodies were raised against AA sequences in the N-CALR and C-CALR domains whose structure is predicted by computational analyses to be in a metastable equilibrium sensitive to environmental cues (Figure 1). For this property, these antibodies may be exploited for epitope mapping studies to identify how the conformation of the protein changes *in vivo* in response to micro-environmental cues as pioneered by the study of Falchi et al. (2017). The resolution of *in vivo* epitope mapping studies is however still low and need to be increased by using panels of antibodies against a wider range of domains of intrinsically disordered and ordered, as internal control, regions of CALR (Figure 1A).

An additional challenge is represented by the ability of CALR to bind numerous proteins. In fact, the immunoglobulins themselves represent such a contamination of samples obtained by immunoprecipitation performed with CALR antibodies that the accurate identification of the additional proteins pulled down by them is almost impossible. Therefore, studies aimed to the identification of the interactome of CALR may need the development of reagents and immunoprecipitation conditions specific for intrinsically disordered proteins.

The intrinsically disordered nature of CALR and the high numbers of partners with which it may be associated also challenge confocal microscopy studies. In fact, because of this caveat, the levels of CALR detected inside a cell by confocal microscopy do not necessary reflect CALR content but may instead indicate the level of exposure of the epitope recognized by the antibody. This is specifically true for antibodies raised against regions of the protein involved in binding with their partners. Therefore, confocal microscopy study should always be performed with multiple antibodies and results confirmed by biochemical studies with purified subcellular compartments.

Last but not least, the intrinsic disordered nature of CALR may also challenge the application of recombinant technologies which have provided a paradigm shift in our way to study protein structure/function relationship. These studies usually involve generation of recombinant genes encoding only part of a protein often linked with a fluorescent tag that may be also recognized by an antibody or a lectin. All these studies are under the assumption that these genetic modifications do not alter the structure and therefore the function of the recombinant protein. These techniques are presently used by many investigators also to study CALR functions. However, as discussed earlier, numerous biophysical studies indicate that the tertiary structure of the individual domains of CALR is strictly dependent on their connections with the other domains. In addition, the recombinant tags, such as the green-fluorescent protein (GFP) used for *in-vivo* imaging or the variety of small, medium and large tags used to study the interactome of a protein [e.g., epitope tags, such as FLAG, six histidines (His)_6_, c-Myc, hemaglutinin (HA), glutathione-S-transferase (GST), protein A (PrA)], utilized in immunoaffinity purification experiments [either individually or in various combinations as is done in a double-tag approach in tandem affinity purification (TAP), or in a triple-tag approach in parallel affinity capture (iPAC)], have all the potential to modify the degree of exposure of individual MoRFs, drastically altering the *in vivo* fate of the recombinant protein and the partners with which it interact.

Biophysical and biochemical studies on wild-type human CALR have been greatly facilitated by the availability of the recombinant protein produced in large quantity in *E. coli* or other expression systems. However, for reasons still unknown, so far attempts to express the forms of CALR lacking the C-domain found mutated in myeloproliferative disorders in *E. coli* have been only partially successful and the mutant proteins are available in minuscule amounts (Ann Mulally, personal communication). This failure unveils the essential role played by the C-domain on the survival of bacteria but also challenges our ability to validate insights on the tertiary structure of the mutated proteins obtained by *in-silico* analyses with experimental approaches.

In conclusion, functional studies of chaperone proteins, such as CALR, by recombinant technology are uniquely challenged by their intrinsically disordered 3D-structure which may be overcome by strict cooperation between and molecular biologists with expert in protein chemistry and biophysics.

## Biological functions of CALR

CALR is involved in many cellular processes, both inside and outside the ER (Wang et al., 2012). In the ER, CALR chaperones nascent unfolded proteins and releases them to the site in which they exert their biological activity and maintains Ca^2+^ homoeostasis. In fact, the C-domain of CALR binds more than 50% of Ca^2+^ present in the ER, thereby functioning as the main Ca^2+^ storage (Nakamura et al., 2001; Michalak et al., 2009). In addition, as a lectin-like molecular chaperone, CALR ensures the proper conformation of newly synthesized glycoproteins, interacting with calnexin and ERp57 in the calreticulin/calnexin cycle, which guides misfolded proteins to the degradation pathway (Michalak et al., 2009). The “folding checking unit” of CALR is represented by its N- and P-domains (Nakamura et al., 2001; Michalak et al., 2009).

Whether CALR is an ER-restricted protein or is also localized in other cellular compartments is an open question subjected to active investigation. CALR may retro-translocate from the ER to the cytosol, where the protein is involved in the regulation of cell adhesion, thanks to a signal sequence present in the C-domain, as well as plays a role in translation, gene expression, and nuclear export (Afshar et al., 2005; Shaffer et al., 2005). In mice, CALR exerts nuclear export function of the nuclear receptors, such as the glucocorticoid receptor (GR) which involves migration to the nucleus (Burns et al., 1994; Holaska et al., 2001). Although, CALR regulates the nuclear export of GR also in human cells, in human cells this activity does not require nuclear localization of CALR but is exerted in regions of the ER located at the nuclear border (Falchi et al., 2017), confirming previous studies which had already indicated that nuclear localization of CALR may be an artifact.

Several investigators have detected cell surface expression of CALR by flow cytometry. On the cell surface, CALR may antagonize the “eat-me” signal provided by CD47 to macrophages. In fact, cell surface up-regulation of CALR has been reported to antagonize phagocytosis of damaged cells during the process of immunogenic cell death (Gardai et al., 2005; Chao et al., 2010). In addition, on the plasma membrane, CALR may function as a receptor for some orphan ligands, such as thrombospondin (Goicoechea et al., 2002). The mechanism responsible for exposing CALR on the cell surface is the subject of active investigation. It is possible that this exposure occurs as part of the continuous remodeling process to which the cellular membranes are subjected.

Last but not least, although there is no report that CALR may be secreted, it is detectable in the extracellular matrix where it may be trapped as part of the degradation process of dead cells. On the extracellular matrix, CALR, may stimulate wound repair and healing by concentrating growth factors, such as Transforming Growth Factor β3, responsible for the proliferation and migration of keratinocytes and fibroblasts to the wound site (Nanney et al., 2008).

## CALR in human stress erythropoiesis

CALR and GR are functionally linked by observations indicating that both protein play important roles in the regulation of the cellular response to a variety of stresses. GR is activated by glucocorticoids secreted by the surrenal gland, which enter the cells passively and binds the receptor in the cytoplasm inducing its dimerization (Zhou and Cidlowski, 2005; Nicolaides et al., 2010). GR dimers bind JAK2, inducing its phosphorylation, and translocate to the nucleus, where they activate the expression of a set of stress-responsive genes by binding to glucocorticoid responsive elements (GRE) present in their regulatory regions (Bauer et al., 1999; Zhou and Cidlowski, 2005). CALR exerts a negative regulation on the cellular response to stress (Dedhar et al., 1994). In fact, studies on murine fibroblasts transfected with GFP-tagged proteins indicate that CALR induces nuclear export of GR, thereby switching off the activation of the stress-responsive genes, and making the cells susceptible to novel ligand activation (Holaska et al., 2001). In these murine cells, this function requires the N-CALR domain, which binds GR (Roderick et al., 1997) and is induced by Ca^2+^ (Holaska et al., 2002), suggesting that is regulated by the conformation of the C-CALR domain (Villamil Giraldo et al., 2010; Wijeyesakere et al., 2011).

In spite of the fact that the hematopoietic system is very sensitive to stress, the functions of CALR in hematopoiesis are still poorly defined. Some indications are starting to emerge on the roles of this protein in the control of stress erythropoiesis, a process of accelerated red cell production activated by anemia induced by hemolysis, blood loss, or oxygen deprivation (Falchi et al., 2017). This process is activated by a combination of high levels of erythropoietin, glucocorticoids, soluble stem cell factor, and bone morphogenetic protein 4 (Erslev and Caro, 1986; Tajima et al., 1998; von Lindern et al., 1999; Millot et al., 2010; Wu and Paulson, 2010). The mechanisms which regulate stress erythropoiesis in humans are studied mostly in *in vitro* models, where hematopoietic progenitors are cultivated in the presence of these growth factors (Varricchio and Migliaccio, 2014). These culture conditions promote the proliferation of a unique class of glucocorticoids-dependent erythroid progenitors (Migliaccio et al., 2011; Xiang et al., 2015) which are capable to upregulate the expression of specific stress responsive genes such as ZFP36L2 which confers them self-replication properties similar to those of hematopoietic stem cells (Liu et al., 2013), thereby allowing the generation of great numbers of red blood cells in a few days (Migliaccio et al., 2002; Varricchio et al., 2011). These culture systems were recently used by us to study the roles of CALR in the stress response of human erythroid progenitor cells (Falchi et al., 2017). This study revealed that in these cells, Ca^2+^ signaling, possibly downstream to the erythropoietin receptor (Misiti and Spivak, 1979), changes the conformation of C-CALR to the state permissive for nuclear export of GR, resetting the stress-responsiveness of the cells and allowing them to undergo the terminal maturation process which had been inhibited by GR activation.

## Abnormalities of CALR in cancer

### CALR mutations found in myeloproliferative disorders

The most evident demonstration for a pathological role of CALR in cancer etiology is provided by the observation that mutations in exon 9 of CALR are found in the Philadelphia-negative myeloproliferative neoplasms essential thrombocythemia and primary myelofibrosis lacking *JAK2*V617F mutations (Klampfl et al., 2013; Nangalia et al., 2013; Nunes et al., 2015). The mutations may be represented either by base pair deletions (Type 1) or insertions (Type 2) and generate a C-CALR domain either shorter or longer than the wild-type one, but lacking in either case the KDEL motif, suggesting that the mutated proteins are not retained in the ER (Figure 1B). In addition to KDEL, the mutations disrupt the low affinity Ca^2+^ binding sites of the protein and three of the putative JAK2-dependent phosphorylation sites, suggesting that mutant proteins may bind Ca^2+^ inefficiently and respond poorly to JAK2 activation. The C-CALR mutants, especially those of type 2, which are characterized by the presence of novel, highly basic C-tails, retain their intrinsically disordered nature, but whether they have lost the ability to affect the overall conformation of the protein, including their effects on the conformation of the N-CALR and P-CALR domains, has not been clarified as of yet.

### Conformation of mutant CALRS found in myeloproliferative disorders

Biophysical studies on protein structure and urea-induced unfolding behavior of mutated CALR similar to those conducted with the wild-type protein are not available as yet. Originally, six different Type 1 and two different Type 2 indel mutations had been identified (Klampfl et al., 2013; Nangalia et al., 2013; Nunes et al., 2015). The list of CALR mutations found in myeloproliferative disorders is however rapidly increasing, and the most recent study described additional 16 Type 1 mutations and three complex indel mutations (Nangalia et al., 2013; Table 1).

**Figure 4:**
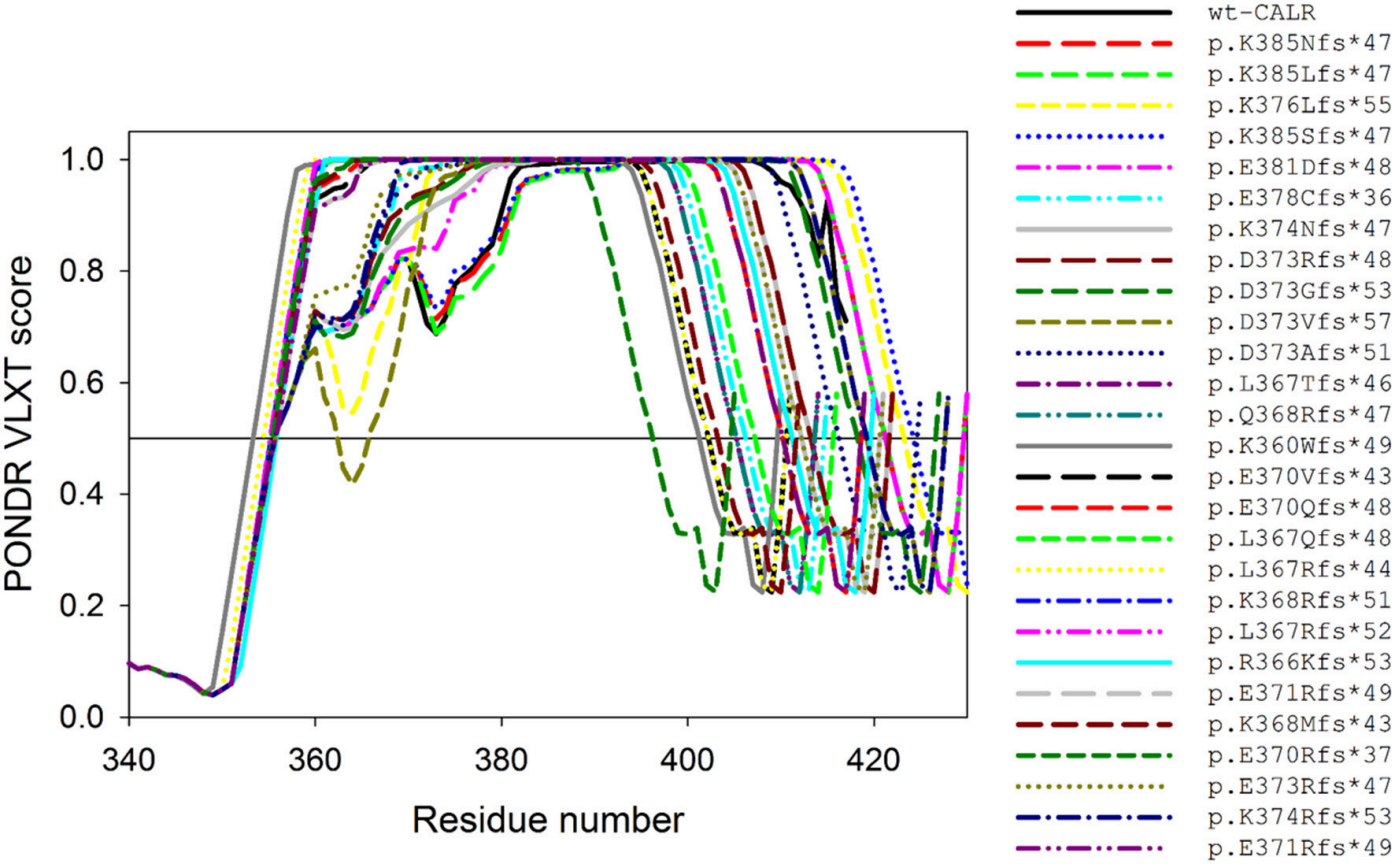
Effects of the indel mutations found in myeloproliferative disorders on the intrinsic disorder predisposition of human CALR. Shown are disorder profiles generated for wild-type CALR and its indel mutations by PONDR® VLXT algorithm (Romero et al., 2001). Since these mutations do not have any effect on the N-CALR, P-domain, and the N-terminal half of C-CALR domain, the plot zooms in the C-terminal part of the C-CALR domain. We used the PONDR® VLXT program because, although is not the most accurate disorder predictor, is sensitive to local peculiarities of the AA sequence and therefore is the program of choice to identify disorder-based potential functional sites. The multiparametic analysis of intrinsic disorder predisposition of wild-type CALR is shown also shown in Figure 2 and is published in Migliaccio and Uversky (2017). See Migliaccio and Uversky (2017).

While the important clinical question of possible correlations between type of mutation and disease phenotype is currently under investigation, attempts to correlate each mutation with its theoretical effects on the disordered nature of CALR are scanty. Still it is highly possible that mutation/phenotype correlations, if they exist, have their basis on differences in protein functions ultimately dictated by its structure. To fill this gap, we conducted preliminary computational analysis, which revealed that the overall disorder status of the C-terminal region of CALR was moderately affected by the indel mutations. Figure 4 presents the disorder profiles generated for wild type CALR and its indel mutations by PONDR® VLXT algorithm (Romero et al., 2001). The changes found in the behavior disorder profiles were rather expected since all the indel mutations possessed identical highly disordered C-tails (last 36 residues), being different from each other by the length of the remaining protein body (ranging from 360 to 385 residues) and the length of the disordered linker region connecting CALR body with this new C-tail (ranging from 0 to 21 residues).

To begin understanding how indel mutations may affect the interactivity of CALR, we also searched their sequence for MoRFs with the ANCHOR algorithm (Dosztányi et al., 2009; Mészáros et al., 2009). To this end, the AA sequence of each mutant protein was analyzed by ANCHOR, and the respective outputs were tabulated (see Table 1). Since indel mutations alter the AA sequence of the C-terminal region of CALR, it was not surprising that all the indel mutations are predicted to affect the two MoRFs (MoRF4 and MoRF5) of the wild-type protein located in the C-terminal half of C-CALR. However, in addition to these somehow expected changes, five of the indel mutations introduce novel MoRF4/5s which may bind novel partners. These findings predict that mutant CALRs, in addition to be loss-of-function mutation by lacking the KDEL motif and low affinity Ca^2+^ binding sites, may be gain-of-function because they lost three putative JAK2-dependent phosphorylation sites and acquired additional MoRFs for novel partners. In spite of all the caveats described in the previous section, studies on the interactome of the various mutant CALRs are badly necessary to validate this hypothesis.

### Alterations of the biological activity of CALR in myeloproliferative disorders

Surprisingly, the phenotype of myeloproliferative disorders associated with *CALR* mutations includes growth factor independent activation of JAK2 (Klampfl et al., 2013; Nangalia et al., 2013), and these patients respond to treatment with JAK inhibitors, such as Ruxolitinib (Cervantes et al., 2013; Passamonti et al., 2014). Studies in human megakaryocytic-erythroleukemic cell lines and in mouse models have elucidated that CALR binds to the cytokine receptor homology domain of all the receptors of the hematopoietic superfamily, including the thrombopoietin (MPL), the erythropoietin, and the G-CSF receptor, and that binding of the mutated, but not wild-type, CALR to MPL, but not to the receptor of EPO or G-CSF, acts as a ligand-mimetic activating JAK2 (Araki et al., 2016; Chachoua et al., 2016). These results have established a common pathological pathway for myeloproliferative disorders caused by JAK2 or CALR mutations.

**Figure 5:**
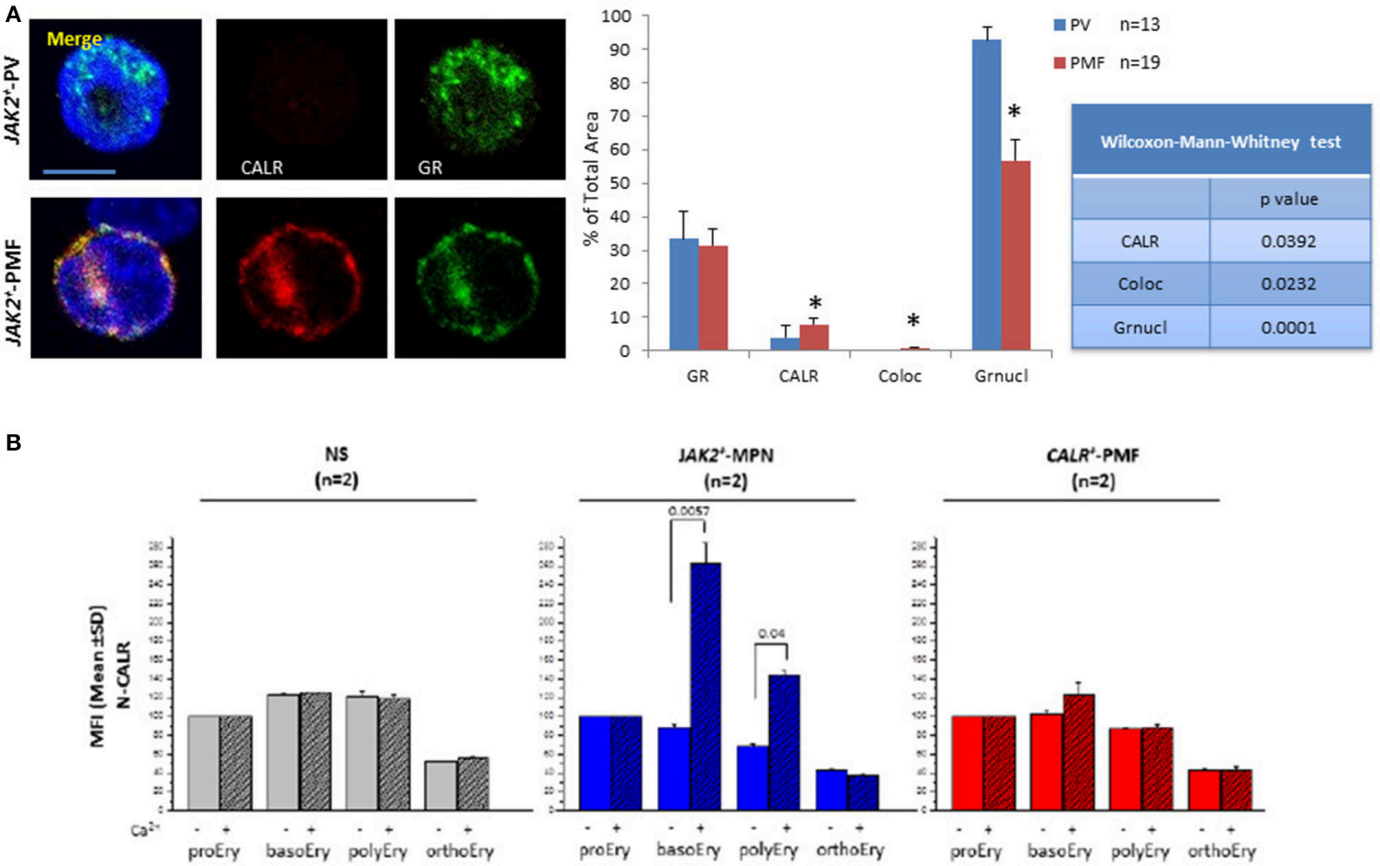
The *JAK2*V617F mutation abrogates the association between CALR and the glucocorticoid receptors in patients with Polycythemia Vera. It also makes the mutant erythroid progenitors capable to up-regulate the cell surface expression of CALR in response to Ca^2+^. **(A)** Single and merged confocal microscopy observations of a representative erythroid progenitor cell expanded in culture from patients with JAK2^+^ Polycythemia Vera (PV) and Primary Myelofibrosis (PMF) stained with antibodies against C-CALR (red) or the glucocorticoid receptor (green), as indicated. Merged areas are in yellow. Nuclei were counterstained in blue with DAPI. Original magnification: 1,200x. The bar corresponds to 10 μm. The results with PV are similar to those published in (Falchi et al., 2017). The results with PMF are not published and were obtained in experiments conducted in parallel with those on PV. Technical details are provided in and are also shown in Figure 2. ^*^Indicates values statistically different (*p* < 0.05) between PV and PMF. **(B)** Levels of N-CALR detected by flow cytometry on the cell surface of erythroid cells expanded *in vitro* from non-diseased donors (ND, 2 different subjects) or from patients with myeloproliferative neoplasms carrying either the *JAK2*V617F or the Type 2 CALR+ mutation (2 patients in each group) and treated *in vitro* for 2 h with EPO in the presence of either Ca^2+^-depleted or Ca^2+^-supplemented phosphate buffered saline. Results are expressed as Mean Fluorescence intensity (MFI) obtained in separate experiments performed in duplicate. Erythroid cells were divided into classes of progressive maturation (proerythroblasts, basophilic, polychromatophilic, and orthochromatic erythroblasts) according to established flow cytometry criteria based on CD36 and CD235a staining (Migliaccio et al., 2011). The levels of cell surface expression of N-CALR between cells exposed or not to Ca^2+^ is statistically different (by Anova) only for erythroid cells harboring the JAK2 mutation. The results of the cells not treated with Ca^2+^ were already published in Falchi et al. (2017). The technical details of these experiments are provided in Falchi et al. (2017).

Conversely, the putative JAK2-dependent phosphorylation sites present in wild-type CALR suggest that in myeloproliferative disorders JAK2 mutations may affect the function of this protein. The observation that in normal erythroid progenitors, Ca^2+^ signaling, possibly downstream to EPO receptor, activates the nuclear GR export functions of CALR inspired us to use this function as a bait to assess whether *JAK2*V617F alters the functions of wild-type C-CALR. This study investigated the nuclear export activity of wild-type CALR in erythroid progenitors from patients with Polycythemia Vera carrying the *JAK2*V617F mutation (Falchi et al., 2017). The results indicated that in contrast with normal cells, in erythroid progenitors from *JAK2*^+^ Polycythemia Vera, C-CALR is not associated and does not induce nuclear export of GR, which remain constitutively active in the nucleus, possibly contributing to their hyper-proliferation (Figure 5A). Therefore, *JAK2*V617F impairs at least the nuclear export activity of CALR. Further studies are necessary to elucidate the full spectrum of CALR functions impaired by *JAK2*V617F, the variegation of these alterations in disorders with different phenotypes and how these alterations are related to those induced by *CALR* mutations.

A peek at possible differences between abnormalities in CALR functions induced by *JAK2*V617F mutations in diseases with different phenotypes is provided by the confocal microscopy observations presented in Figure 5A. These data indicate that the CALR/GR association is impaired and nuclear retention of GR is abnormally high in *JAK2*V617F patients expressing both the Polycythemia Vera and the Primary Myelofibrosis phenotype. By contrast with Polycythemia Vera patients who experience erythrocytosis, patients with Primary Myelofibrosis express anemia which is not always responsice to erythroid-stimulating agents. The observation that erythroid progenitor cells from Primary Myelofibrosis retain some level of CALR/GR association displaying more GR in the cytoplasm than those from Polycythemia Vera suggests that these cells may be at least partially responsive to glucocorticoids and may explain anectodical reports that indicate that a fraction (3 out of 10) of Primary Myelofibrosis patients resistant to classic erythroid-stimulating agents (Hernández-Boluda et al., 2017) are cured of their underlying anemia and became transfusion-independent when treated with glucocorticods (G. Barosi, personal communication, May 2017). The reason why only some of the Primary Myelofibrosis patients respond to glucocorticoids is still to be determined. The inhibitory effect exerted by JAK2V617F on nuclear export of GR described above suggests that patients with high *JAK2*V617F allele burden are those that became glucocorticoids non responsive. Further studies are necessary to test this hypothesis.

Support for the hypothesis that the JAK2 mutation may affect CALR functions is further provided by the flow cytometry study presented in Figure 5B. By flow cytometry, CALR is readily detected on the cell-surface of normal erythroid progenitors and remain expressed in cells undergoing terminal maturation up to the polycromatophilic stage (Figure 5B). In these normal cells, cell-surface expression of CALR is not affected by Ca^2+^. Erythroid cells from JAK2V617F myeloproliferative disorders express cell-surface levels of CALR similar to those expressed by normal cells. This observation is consistent with the recent report indicating that CALR is expressed within normal ranges by a variety of hematopoietic cells from patients with the myeloproliferative neoplasm Essential Thrombocythemia (Daitoku et al., 2016). By contrast with normal cells, however, Ca^2+^ upregulates the levels of CALR expressed on the surface of erythroid cells from *JAK2*^+^ patients but not, as expected, in those from patients harboring *CALR* mutations with an impaired C-CALR domain which are unable to bind Ca^2+^ (Figure 5B), suggesting that under certain conditions anti-CD47/CALR therapies (see later) are likely to be effective in myeloproliferative neoplasms. In addition, several groups are currently investigating whether, by altering the intrinsically disordered nature of the protein, these driver mutations induce the appearance of novel, possibly immunogenic, CALR epitopes to be used to generate immune-specific therapies.

### Alterations of the biological activity of CALR in other hematological and solid tumors

CALR is involved in the etiology not only of myeloproliferative disorders but also in that of many other cancers (Zamanian et al., 2013). By contrast with myeloproliferative neoplasms, driver mutations in *CALR* have not been associated with other hematological or solid tumors as yet. However, at least 114 single nucleotide polymorphism sites (SNPs) distributed along the exons have been described as somatic mutations of the *CALR* gene (located on human 19p13.13) in a variety of cancers (see https://hive.biochemistry.gwu.edu/biomuta, UniProt_AC P27797, Accession Number: NM_004343). The curated SNPs and disease association database reports that somatic *CALR* SNP variants are present in patients with at least 20 different types of hematologic malignancies and solid cancers, suggesting that they increase the susceptibility of normal cells to develop driver mutations (Lundberg et al., 2014; Malcovati et al., 2014). Among these 114 mutations, 66 are non-synonymous variations, 40 of which are unique substitutions which have either benign (22 SNPs) or possibly damaging (16 SNPs) consequences on disease phenotype (Table 2). These SNPs are distributed along all of the functional domains of CALR: one SNP is found in the signal peptide, 14 in N-CALR, 6 in P-CALR, and 19 in C-CALR, respectively. Most SNPs have as predicted biochemical consequence gain of phosphorylation sites but gain of glycosylation and loss of binding sites have also been described. The little information on the biological relevance of post-translational modification of CALR currently available limits our understanding of the contribution played by alterations of the post-translational modification sites of CALR in the etiology of cancer.

**Table 2:** Effects of different somatic SNPs most commonly found in cancer on the intrinsic disorder-based interactivity of human CALR.

**SNPs**	**AA Substitutions**	**Predicted function**	**MoRF1**	**MoRF2**	**MoRF3**	**MoRF4**	**MoRF5**
**wt**	**N/A**	**Wild type CALR**	**170–176**	**198–204**	**259–269**	**304–352**	**409–417**
13	V5M	–	170–176	198–204	259–269	304–352	409–417
79	F27L	–	170–176	198–204	259–269	304–352	409–417
133	D45Y	–	170–176	198–204	259–269	304–352	409–417
137	F46Y	Gain/Phosphorylation	170–176	198–204	259–269	304–352	409–417
151	L51V	–	170–176	198–204	259–269	304–352	409–417
168	F56L	Gain/Phosphorylation	170–176	198–204	259–269	304–352	409–417
186	K62N	Gain/Phosphorylation	170–176	198–204	259–269	304–352	409–417
185	K62R	Gain/Phosphorylation	170–176	198–204	259–269	304–352	409–417
191	K64T	Loss/Acetylation	170–176	198–204	259–269	304–352	409–417
201	Q67H	–	170–176	198–204	259–269	304–352	409–417
298	E100Q	Gain/Phosphorylation	170–176	198–204	259–269	304–352	409–417
365	M122T	Gain/Phosphorylation	170–176	198–204	259–269	304–352	409–417
383	Y128C	Loss/Binding Site	170–176	198–204	259–269	304–352	409–417
394	F132L	Gain/Phosphorylation	170–176	198–204	259–269	304–352	409–417
562	N188D	–	170–176	198–204	259–269	304–352	409–417
610	P204S	–	170–176	198–205	259–269	304–352	409–417
700	E234K	Gain/Phosphorylation	170–176	199–205	263–269	304–352	409–417
720	E240D	Gain/Phosphorylation	170–176	198–204	259–269	304–352	409–417
731	D244G	–	170–176	198–204	259–269	304–352	409–417
892	E298Y	–	170–176	198–204	257–296		
899	S300Y	–	170–176	198–204	263–268	303–350	409–417
925	D309Y	Gain/Phosphorylation	170–176	198–204		304–350	409–417
968	S323F	–	170–176	198–204	259–268	304–350	409–417
1003	D335N	–	170–176	198–204	259–269	304–352	409–417
1022	E341G	Gain/Glycosylation	170–176	198–204	259–269	304–352	409–417
1028	G343D	–	170–176	198–204	259–269	304–352	408–417
1103	K368M	–	170–176	198–204	259–269	304–352	408–417
1113	E371D	–	170–176	198–204	259–269	304–352	408–417
1121	K374R	–	170–176	198–204	259–269	304–352	409–417
1137	E379D	–	170–176	198–204	259–269	304–352	408–417
1142	E381G	–	170–176	198–204	259–269	304–352	409–417
1157	E386G	–	170–176	198–204	259–269	304–352	409–417
1167	E389D	–	170–176	198–204	259–269	304–352	408–417
1192	E398K	–	170–176	198–204	259–269	304–352	409–417
1207	E403Y	–	170–176	198–204	259–269	304–350	
1212	D404E	–	170–176	198–204	259–269	304–352	409–417
1213	E405Q	–	170–176	198–204	259–269	304–352	408–417
1229	P410L	–	170–176	198–204	259–269	304–351	406–417
1240	K414E	–	170–176	198–204	259–269	304–352	408–417
1245	D415E	–	170–176	198–204	259–269	304–352	409–417

*Information on SNP identification number (column 1) and description of the related AA substitution (columns 2), as well as predicted functional outputs (column 3) were taken from https://hive.biochemistry.gwu.edu/biomuta. Expected effects of SNPs on the interactivity of CALR were evaluated by the ANCHOR algorithm (Dosztányi et al., 2009; Mészáros et al., 2009). To this end, the AA sequence of each mutant identified in column 2 was analyzed by ANCHOR and the corresponding outputs (positions of MoRFs) were tabulated. SNPs with possibly/probably damaging effects on disease phenotype are indicated in red. MoRFs alterations caused by SNPs are highlighted in yellow*.

**Figure 6:**
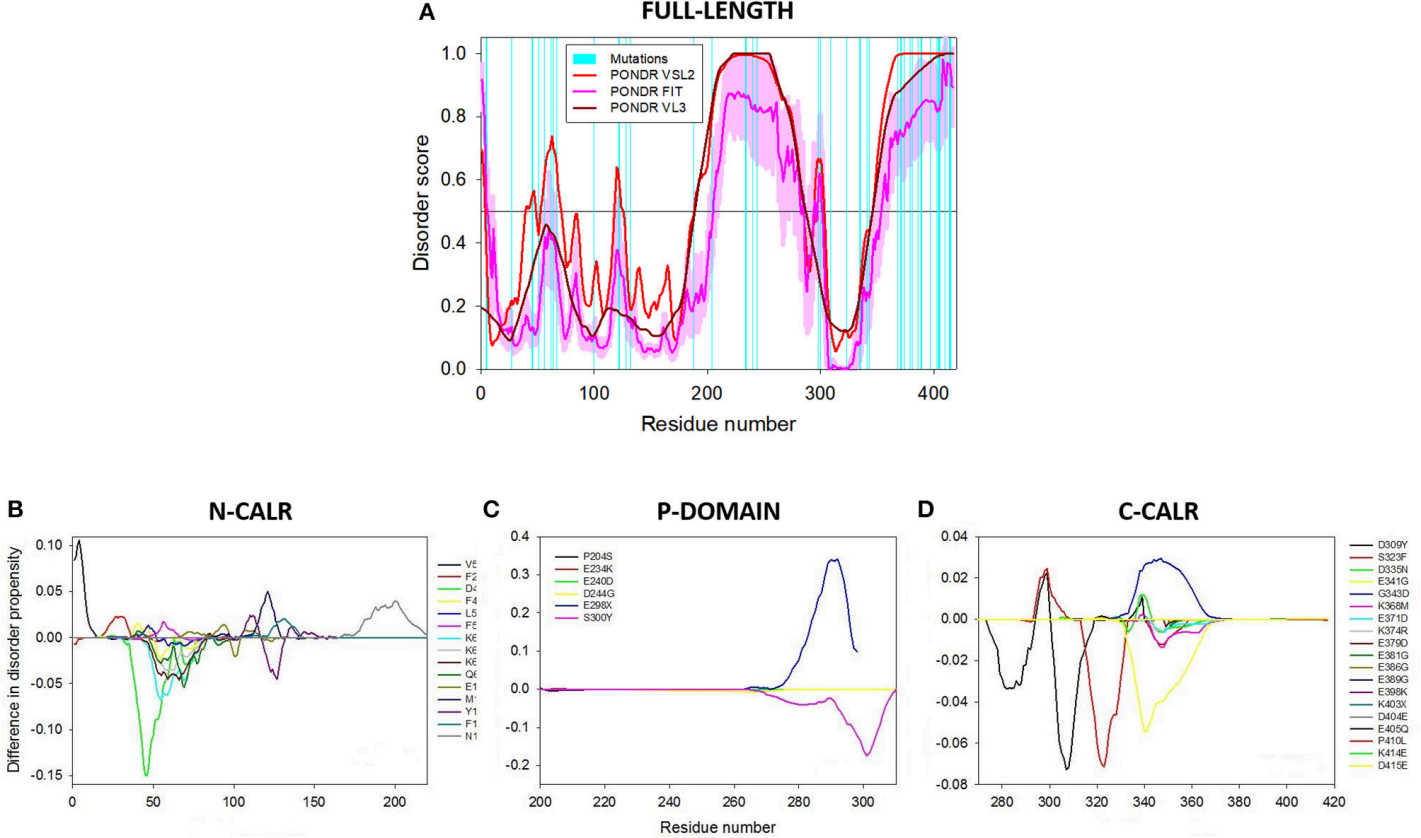
Effects of somatic single-nucleotide polymorphisms (SNPs) of CALR detected in cancer patients on the intrinsic disorder nature of the protein. **(A)** Intrinsic disorder propensity of human CALR evaluated by PONDR® VSL2 (red), PONDR® VL3 (dark red), and PONDR® FIT (pink). PONDR® VSL2 is a rather accurate stand-alone disorder predictor suitable for proteins containing ordered and intrinsically disordered regions. PONDR® VL3 is developed for accurate prediction of long disordered regions. Metapredictor PONDR® FIT is one of the more accurate disorder predictors. Position of 40 unique non-synonymous mutations are shown by cyan bars. Light pink shadow around the PONDR® FIT shows error distribution. In these analyses, predicted intrinsic disorder scores above 0.5 are considered to correspond to the disordered residues/regions, whereas regions with the disorder scores between 0.2 and 0.5 are considered flexible. **(B–D)** “Difference disorder spectra” calculated as a difference between the per-residue disorder propensity evaluated by the PONDR® VSL2 for the mutant forms of human CALR and the per-residue disorder propensity evaluated by the PONDR® VSL2 for the wild-type protein. The protein sequence is split into three parts to zoom into the N-CALR **(B)**, the P-CALR **(C)**, and the C-CALR **(D)** domain. The multiparametic analysis of intrinsic disorder predisposition of wild-type human CALR was published in Migliaccio and Uversky (2017; see also Figure 2). See Migliaccio and Uversky (2017).

The vast majority of these SNPs are located either in disordered or flexible regions (i.e., regions characterized by the predicted disorder scores PDS ≥ 0.5 or 0.2 ≤ PDS < 0.5, respectively). This is an interesting observation that further supports the hypothesis that these intrinsically disordered/flexible regions play an important role in the functionality of human CALR. To start delineate the effects of these SNPs on the tertiary structure of CALR, we used a set of commonly used disorder predictors such as PONDR® FIT (Xue et al., 2010), PONDR® VSL2 (Obradovic et al., 2005), and PONDR® VL3 (Peng et al., 2005) to predict the effects of some of these SNPs on the disorder profiles of the human protein (Figure 6A). The effects on the intrinsic disorder predisposition of specific domains was instead determined by the PONDR® VSL2 predictor (Obradovic et al., 2005) in a form of “disorder difference spectra” that were calculated as a simple difference between the PONDR® VSL2 disorder curves calculated for variant and wild-type forms of CALR (Figures 6B–**D**). Obviously, in this presentation, negative or positive peaks correspond to SNPs leading to local decrease or increase in the intrinsic disorder propensity, respectively. Since CALR contains numerous SNPs, to show effects of each variant in more detail, results corresponding to the N-CALR, P-domain, and C-CALR domains are presented in different panels. Figure 6 shows that many SNPs have profound effects on the local disorder propensity of this protein. The largest distortion in the local disorder predisposition was found for variants located within the more ordered N-CALR domain. In fact, all mutations in this region caused at least some changes in the local disorder propensity. Also, mutations within the more ordered C-terminal part of the P-domain (E298X and S300Y) and N-terminal part of C-CALR (D309Y, S323F, E341G, D335N, and G343D) caused noticeable alterations in the local disorder predisposition, whereas local disorder propensities of the highly disordered parts of the P-domain and C-tail of C-CALR were either not altered at all (mutations P204S, E234K, and E240D in P-domain and E379D, E381G, E386G, E389G, E398K, K403X, D404E, E405Q, P410L, K414E, and D415E in C-CALR) or experienced rather minor distortions (e.g., mutations K368M, E371D, and K374R; see Figures 6B–D).

We also analyzed how pathological point mutations might affect disorder-based potential binding sites of CALR by subjecting sequences of its mutants to ANCHOR analysis (Dosztányi et al., 2009; Mészáros et al., 2009). The analysis is summarized in Table 2 and indicates that MoRF1 was not affected by any variants. However, MoRF2 was extended by 1 residue by P204S, MoRF3 was reduced by 4 residues by E234K (264–269). In addition, E298X introduced a premature stop codon and removed the C-tail with MoRF4 and MoRF5, S300Y affected MoRF3 (263–268) and MoRF4 (303–350), D309Y eliminated MoRF3 and changed MoRF4 (304–350), S323F mutation changed MoRF4 (304–350); G343D, K368M, E371D, E379D, E389D, E405Q, and K414E each extended MoRF5 by 1 residue (408–417), K403X introduced a premature stop codon, removed the C-tail with MoRF5 and shrunk MoRF4 by two residues (304–350), P410L decreased MoRF4 by one residue (304–351) and extended MoRF5 by three residues (406–417). Overall, these analyses showed that most of the clinically relevant SNPs affect MoRF3 and/or MoRF5. Therefore, it is conceivable that at least some of these changes alter the interactome of CALR. These predictions based on computer-modeling must be confirmed by additional biophysical and biochemical studies similar to those discussed earlier for the wild-protein.

In general, the biological consequences of somatic CALR SNPs associated with cancer are far less characterized than those of the mutant proteins found in myeloproliferative neoplasms. The major CALR defect described so far in cancer cells regards the levels of cell-surface expression. CALR is expressed at abnormally high levels on the cell-surface of a wide variety of cancer cells, including some hematological malignancies (Wemeau et al., 2010; Zamanian et al., 2013, 2016), suggesting that, by antagonizing the “eat-me” signal provided by CD47, CALR may promote disease progression via inhibition of the surveillance by the immune system (Obeid et al., 2007; Chao et al., 2010). These observations are guiding the development of drugs that target the CD47/CALR pathway as a way to treat cancer by reactivating the immune surveillance system (Chao et al., 2011; Majeti, 2011; Kong et al., 2016). This is such a potentially important area of research that deserves to be further investigated.

## Conclusions

CALR is emerging as a structural “chameleon” protein with multiple structural facets, each one involved in specific functions. Mutations affecting the integrity of its Ca^2+^-binding domain alters the proliferation of hematopoietic cells at levels that induce myeloproliferative neoplasms. In addition, somatic mutations distributed along all the sequence of the gene with the potential to determine fine differences in protein activity are found in many cancers. These CALR variants may represent genetic modifiers that facilitate the development of driver mutations and/or alter the natural history of the disease. Hence, the great clinical interest to develop novel prognostic/therapeutic tools by gaining a deeper understanding on the biological function of CALR. The intrinsic disordered nature of this protein, however, is such that it may be a mistake to consider CALR as just one protein. This hypothesis and theory article has discussed how each one of its alternative conformational ensembles may exert specific functions and challenge future studies on the association of a specific ensemble with a specific function in the next few years.

## Author contributions

AM wrote the manuscript. LV, MF, MD, CD, AR, VU contributed to write the manuscript and revision.

### Conflict of interest statement

The authors declare that the research was conducted in the absence of any commercial or financial relationships that could be construed as a potential conflict of interest.
